# Autoimmune Encephalopathy Associated With Anti-thyroid Antibodies: A Case Report

**DOI:** 10.7759/cureus.28183

**Published:** 2022-08-19

**Authors:** Vladimir Falb, Louis Costanzo, Cesar Avalos, Aleksander Feoktistov

**Affiliations:** 1 Internal Medicine, State University of New York Downstate Medical Center, Brooklyn, USA; 2 Rheumatology, State University of New York Downstate Medical Center, Brooklyn, USA

**Keywords:** anti-thyroglobulin, sreat, catatonia, thyroid peroxidase antibody, hashimoto’s encephalopathy

## Abstract

Steroid-responsive encephalopathy associated with autoimmune thyroiditis (SREAT), also known as Hashimoto encephalopathy (HE), is a rare condition. HE is characterized by abnormal brain function associated with elevated titers of anti-thyroid peroxidase (anti-TPO) and/or anti-thyroglobulin (anti-Tg) antibodies. We present a case of a 19-year-old female with rapidly progressing psychosis with mutism, catalepsy, echopraxia, and catatonia that developed over the course of three months. She was found to have high-level anti-thyroid antibodies raising suspicion of subclinical autoimmune thyroiditis and positive antinuclear antibodies. Imaging of the brain revealed generalized cerebral atrophy abnormal for her age. The patient was aggressively treated with corticosteroids and immunomodulators and her symptoms were greatly improved. This case emphasizes the significance of thyroid antibody measurement in patients presenting with psychiatric symptoms to evaluate patients for autoimmune encephalitis, since treatment with steroids and other immunosuppressive agents may be warranted.

## Introduction

Hashimoto encephalopathy (HE), or steroid-responsive encephalopathy associated with autoimmune thyroiditis (SREAT), is an uncommon disease with a varying presentation. SREAT is typically present after the fourth decade of life and is more common in women. The disease causes significant progressive cognitive decline but may be relapsing and remitting [1]. The pathogenesis of SREAT is widely disputed and unclear, as it may result from direct antibody-mediated neuronal injury [2], endothelial inflammation [3], or immune complex deposition [4]. However, HE should be suspected in patients with elevated anti-thyroid peroxidase (anti-TPO) antibodies and/or anti-thyroglobulin (anti-Tg) antibodies presenting with rapidly progressing psychosis and neurologic dysfunction. We report a case of a young adult female who had multiple hospital visits for varying presentations from psychosis to catatonia. She was ultimately found to have elevated anti-TPO antibodies suggesting autoimmune thyroiditis and was diagnosed with SREAT and successfully treated with corticosteroids and immunomodulating agents.

## Case presentation

A 19-year-old female with a past medical history of mood disorder and type 2 diabetes was brought to the hospital with complaints of five days of increased thirst, frequent urination, fatigue, slow speech, and an elevated finger-stick glucose measurement of more than 500 mg/dL.

The patient was hospitalized multiple times over the course of three months for progressively worsening psychosis, mutism, catalepsy, and echopraxia; and most recently for acute onset psychosis requiring a longer stay. During her recent hospitalization, blood tests revealed positive antinuclear antibodies of 1:640 and positive anti-TPO antibodies with normal thyroid-stimulating hormone (TSH) (2.5 mIU/L) and T4 (7 mcg/dl). Her last hospital admission one month ago was complicated by catatonia, for which she underwent treatment with 13 cycles of electroconvulsive therapy resulting in minimal improvement. However, it was determined that the patient may have possible encephalitis given her psychiatric symptoms and positive antibodies, which were responsive to treatment with prednisone 40 mg and intravenous immunoglobulin (IVIG). She was discharged with a prednisone taper, prophylactic pantoprazole, mycophenolate mofetil, and continued IVIG therapy. Since discharge, she has continued to follow-up with psychiatry and rheumatology. Rheumatology recommended continuing with prednisone 5 mg daily and mycophenolate mofetil 1000 mg orally twice per day. 

Medications at the time of admission were prednisone (taper), pantoprazole 20 mg daily, aripiprazole 30 mg daily, clozapine 125 mg daily, lorazepam 2 mg three times per day, mycophenolate mofetil 1000 mg twice daily, and vitamin D3 1000 IU. She had no recent surgeries, travel, or trauma, and did not take any illicit substances.

**Figure 1:**
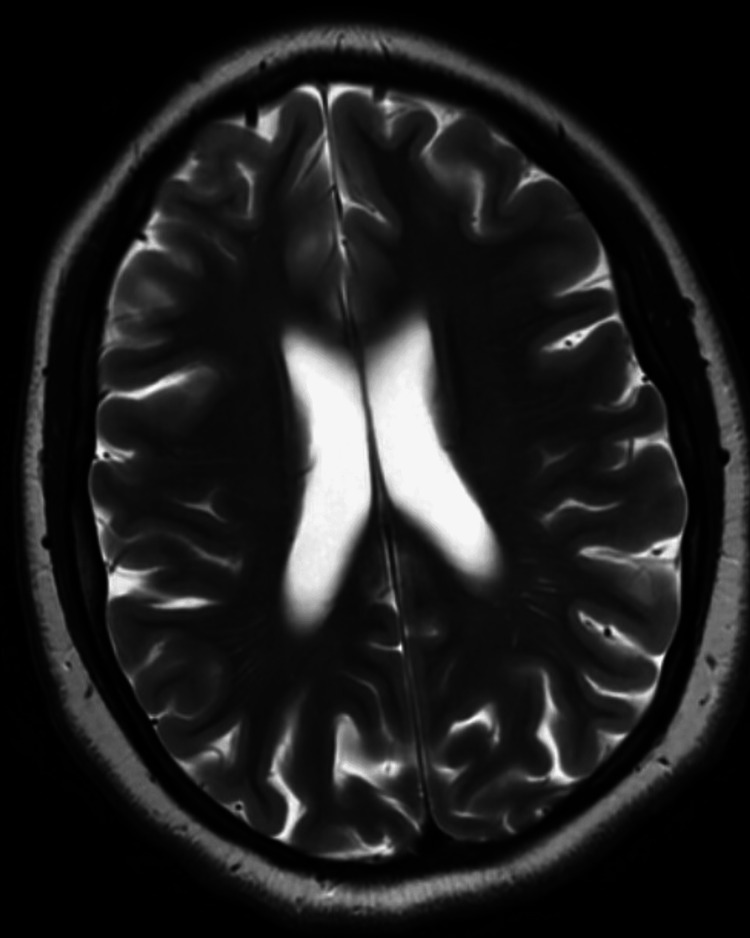
Abnormal cerebral atrophy. Magnetic resonance imaging (MRI) depicting generalized cerebral atrophy with the prominence of the sulci, fissures, and ventricles, abnormal for the patient’s age.

Her vital signs showed a blood pressure of 130/80 mmHg, heart rate of 70 beats per minute, respiratory rate of 12 breaths per minute, and oxygen saturation of 98%. Physical and psychiatric exams revealed a flat affect with bradylalia, multiple striae, and a prominent dorsocervical fat pad. Laboratory testing revealed a blood glucose level of 410 mg/dL with an anion gap of 18 and a urinalysis positive for ketonuria and glycosuria. Thyroid function tests showed a thyroid-stimulating hormone of 1.04 and a free T4 of 1.2. A chest x-ray did not show any acute abnormalities. A previous magnetic resonance imaging (MRI) brain one month ago showed mild generalized cerebral atrophy, abnormal for the patient’s age (Figure 1). 

Given her history and clinical presentation, a lumbar puncture was performed during this admission. The cerebrospinal fluid revealed a WBC count of 3 cells/mcL, protein 30.1 mg/dL, glucose 60 mg/dL, absent oligoclonal bands, and the following negative autoimmune (ENC1) evaluation: N-methyl-D-aspartate-receptor antibodies (anti-NMDAR encephalitis), LGI1-IgG antibodies (limbic encephalitis), contactin-associated protein-like-2 receptor antibodies (autoimmune epilepsy, limbic encephalitis, Morvan syndrome), glutamic acid decarboxylase 65 antibodies (neurologic-associated autoimmune disease), anti-glial nuclear antibodies (Lambert-Eaton syndrome, paraneoplastic neurologic disorders), γ-aminobutyric-acid-B receptor antibodies (limbic encephalitis), α-amino-3-hydroxy-5-methyl-4-isoxazolepropionic acid receptor antibodies (limbic encephalitis), anti-neuronal nuclear antibodies types 1-3 (paraneoplastic neurologic disorders, encephalomyeloradiculopathies), anti-glial-nuclear antibodies type 1 (paraneoplastic neurologic disorders), Purkinje cell cytoplasmic antibodies types 1-2 and Tr (paraneoplastic cerebellar degeneration), amphiphysin antibodies (Stiff-Person syndrome, paraneoplastic neurologic disorders due to small cell lung carcinoma or brain tumors), collapsin-response-mediator-protein 5 IgG antibodies (paraneoplastic neurologic disorders), dipeptidyl-peptidase-like-protein antibodies (CNS hyperexcitability, various types of encephalitis), glial fibrillary acidic protein antibodies (astrocytic cell marker of meningoencephalitis or meningoencephalomyelitis) mGluR1 antibodies (autoimmune cerebellar ataxia), and 14-3-3 protein levels (Creutzfeldt-Jakob disease).

The patient was given intravenous fluids and insulin. Rheumatology recommended the continuation of prednisone 10 mg daily, mycophenolate 1 g twice daily, and IVIG 0.4 g/kg/day for two days. The patient was also seen by neurology, who did not recommend any neurological inpatient intervention at this time. Psychiatry had seen the patient as well and recommended a lorazepam taper. The endocrinology recommendation was to continue Novolin 70/30 mix, 25 U AM, and 15 U PM, given an HbA1c of 10.7%. However, the patient did not show any improvement with the current regimen; therefore, daily intravenous methylprednisolone 1000 mg was started. After a three-day course, her clinical status greatly improved, and she was ultimately discharged with appropriate follow-up.

## Discussion

Hashimoto encephalopathy (HE) was first described in 1966 in an elderly patient with hemiparesis and biopsy-confirmed Hashimoto’s thyroiditis [5]. Additionally, Shaw et al. documented five separate cases of neurologic symptoms associated with positive thyroid antibodies in the early 1990s [6]. Since then, several papers have described signs of encephalopathy and elevated thyroid antibody levels; referring to HE as “myxedema madness” [7], “encephalopathy associated with autoimmune thyroid disease” [8], or “steroid-responsive encephalopathy with antibodies to thyroperoxidase”, or SREAT [9]. However, there is limited documentation on the pathogenesis, diagnosis, and treatment of this uncommon condition.

Pathogenesis of SREAT

The pathogenesis of SREAT is not well understood. Although anti-thyroid antibody positivity is important for the diagnosis of SREAT, its role in the underlying pathogenesis mechanism remains unclear, and no direct correlation between serum antibody titers and the clinical state of disease severity is found. Given the wide range of symptoms associated with SREAT, pathophysiologic mechanisms that underlie SREAT may include disseminated encephalomyelitis [10], as well as cerebral vasculitis [11] resulting in a broad spectrum of multifocal neurologic abnormalities [12]. It has been suggested, however, that direct action of thyrotropin-releasing hormone (TRH) may lead to neurologic deficits as neurologic symptoms increase with the infusion of TRH [13]. Additional ideas regarding SREAT pathogenesis refer to the direct binding of anti-TPO antibodies to astrocytes in the central nervous system, which contributes to a myriad of neurologic symptoms, further emphasizing a correlation between SREAT and autoimmune thyroiditis [14]. In addition, the pathogenic roles of antibodies in SREAT have been questioned. Rather than playing a direct role in the pathophysiology of SREAT, it is suggested that thyroid-associated anti-TPO may be a biologic marker of SREAT [15]. Furthermore, Laurent et al. studied 251 patients with pre-existing thyroiditis who presented with signs of encephalitis and determined that serum anti-TPO antibodies were positive in greater than 30% of patients [16].

Pathophysiologically, SREAT belongs to a group of rare heterogeneous disorders described as autoimmune encephalitis (AE) characterized by an immune-mediated response to various self-antigens expressed in the central nervous system inflammation of the brain [17]. This autoimmune condition often involves the cortical or deep gray matter, but inflammation can rarely occur within the white matter and meninges [18].

SREAT and elevated thyroid antibodies

It is important to note that anti-TPO antibodies are frequently present in 12-26% of euthyroid patients [19] and detected in approximately 13% of individuals within the general population [20]. Diagnostic criteria for Hashimoto’s thyroiditis, an autoimmune disorder characterized by decreased thyroid hormone production, consists of positive anti-thyroid antibodies, which early in the disease may be in the presence of normal TSH and free T4.

Autoimmune encephalitis has been associated with autoimmune thyroiditis. Patients with autoimmune encephalitis, especially those with SREAT, usually exhibit elevated titers of anti-TPO antibodies [21,22]. Moreover, SREAT has more recently been characterized in the literature as neurologic dysfunction exhibited in patients with high titers of anti-thyroid peroxidase antibodies and anti-thyroglobulin antibodies [23]. However, this does not mean that all patients with SREAT have additional thyroid hormone abnormalities. In fact, anti-TPO antibodies were found to be high in pediatric patients with SREAT; with normal TSH and T4 titers [24]. Therefore, anti-TPO may serve as a marker of autoimmunity rather than a disease-specific or pathogenic antibody [25]. However, the presence of anti-TPO antibodies or additional nonspecific thyroid antibodies should prompt clinicians to consider SREAT in patients with suggestive signs and symptoms.

Clinical presentation and diagnosis of SREAT

Given its rarity and a broad range of symptoms, SREAT may be overlooked or mistreated, as it has a subacute onset and progresses quickly over one to three months [26]. Symptoms of SREAT in this context are usually nonspecific but may include impaired memory and understanding, new focal neurologic deficits, facial dyskinesia, unusual and involuntary movements, new-onset seizures, and catalepsy. In the case of our patient, rapidly progressing symptoms such as mutism, catalepsy, and echopraxia were present, as well as catatonia, the latter of which has been linked to various types of autoimmune encephalitis [27]. However, it is also important to note that SREAT can present acutely [28] and in a relapsing-remitting manner, including cognitive deterioration and psychosis [29]. 

The diagnosis of autoimmune encephalitis can be considered when the following three symptoms are met: (a) less than three months of memory deficits, altered mental status, or psychiatric symptoms; (b) new focal neurologic deficits, unexplained new-onset seizures, CSF pleocytosis, or MRI features suggestive of AE (often bilateral hyperintense T2/fluid-attenuated inversion recovery (FLAIR) abnormalities in the basal ganglia and brain atrophy that may spare the temporal lobes); and (c) reasonable exclusion of other possible causes of the condition [30]. Given the wide range of symptoms manifesting in SREAT specifically, the presence of anti-TPO antibodies should prompt the consideration of SREAT. Various reports, such as that from Chong et al., concluded that the diagnosis of SREAT in 105 cases was frequently based on altered consciousness, negative infectious workup, and high levels of anti-thyroid antibodies, the latter of which was found in 100% of cases [31]. Furthermore, anti-thyroglobulin antibodies, CSF abnormalities including lymphocytic pleocytosis with oligoclonal bands, nonspecific EEG activity, and focal white matter changes on MRI may aid in the diagnosis of SREAT [32]. Clinical suspicion should remain high in patients with elevated anti-TPO antibodies in the setting of the appropriate clinical picture.

Treatment of SREAT

Treatment of AE involves the administration of corticosteroids and aggressive immunosuppression. Oral or intravenous (IV) corticosteroids may be given. A course of IV methylprednisolone 30/mg/kg per day was reported to be given to multiple patients with SREAT followed by a course of oral prednisone, and all patients returned to their clinical baseline [33]. Our patient’s clinical status dramatically improved after treatment with 1000 mg of IV methylprednisolone for three daily infusions. In fact, a review by Laurent and colleagues in which 193/203 patients received corticosteroids as first-line therapy for SREAT, suggested that the majority of patients showed complete or partial neurological responses 12 months later with decreasing anti-TPO antibody titers after treatment, which included 1 g of IV methylprednisolone followed by oral prednisone 60 mg per day 15. In addition, intravenous immunoglobulin (IVIG) at 2 mg/kg has been successfully used in patients not responding to steroids [34]. Plasmapheresis can also be considered second-line treatment. Additionally, immunosuppressive agents such as mycophenolate, methotrexate, and azathioprine can be treatment options and have been associated with a reduction in anti-thyroid antibodies and improved neurologic symptoms [35]. 

## Conclusions

Hashimoto encephalopathy (HE), or SREAT, is a rare entity associated with increased anti-thyroid antibodies and autoimmune thyroiditis. Clinicians should have increased suspicion in patients presenting with neurologic and mood symptoms with elevated anti-thyroid antibody levels. The diagnosis can be considered when the patient exhibits rapidly progressing cognitive decline, a negative infectious workup, and a high level of anti-thyroid antibodies. Once SREAT is suspected, aggressive treatment with corticosteroids and immunomodulators can lead to significant improvement in symptoms. IVIG can also be considered.

It is important for clinicians to remember that anti-TPO antibodies are associated with various autoimmune disorders. Not only are anti-TPO antibodies an early predictive marker for thyroid disease, but anti-TPO antibodies have been linked to extra-thyroidal diseases such as vitiligo, orbitopathy, and autoimmune encephalitis. In the setting of an appropriate clinical picture and elevated anti-TPO antibody levels, an extensive evaluation should be pursued to evaluate for autoimmune-related conditions, especially Hashimoto encephalopathy.
